# Policy perspectives and attitudes towards mental health treatment in rural Senegal

**DOI:** 10.1186/1752-4458-8-9

**Published:** 2014-03-19

**Authors:** Nicole M Monteiro, Youssoupha Ndiaye, Demetri Blanas, Idrissa Ba

**Affiliations:** 1 Department of Psychology, University of Botswana, Private Bag, Gaborone 00775, Botswana; 2 Ministry of Health and Social Action, Dakar, Senegal; 3 Institute For Family Health/Icahn School of Medicine at Mount Sinai, New York, USA; 4 Faculté de Médecine, Pharmacie et Odontostomatologie, Université Cheikh Anta Diop de Dakar, Dakar, Senegal

**Keywords:** Sub-Saharan Africa, Senegal, Mental health, Primary care system, Traditional healing

## Abstract

**Background:**

Mental health is often given low priority in health policy planning, particularly in developing countries. Several international health bodies, including the World Health Organization, recommend integrating mental health into primary care settings to reduce mortality and morbidity associated with mental illness, particularly in low-resource settings.

**Objective:**

This study explores health care workers' and policy stakeholders’ knowledge and attitudes regarding mental illness, interactions with patients in the community, and perceived training needs at a health clinic in rural southeastern Senegal. Interviews were conducted with eight key informant medical staff members and community health workers.

**Methods:**

Interview data were analyzed and interpreted using a qualitative content analysis based on the grounded theory approach.

**Results:**

The findings indicate that staff members encounter many patients with emotional/psychological problems or mental illnesses, and they employ various strategies in treating these patients. Respondents also highlighted the need for more training to address and diagnose mental health problems, especially severe psychiatric illnesses.

**Conclusions:**

Findings are used to discuss recommendations for developing a comprehensive mental health primary care treatment approach that includes screening patients for mental health problems screening, incorporating rural villagers' attitudes and beliefs about mental illness into treatment, and utilizing community health workers—who are often a first health contact for many—to work with the medical staff to identify mental health problems.

## Background

Numerous studies and policy reports have underscored the importance of integrating mental health into primary health care in resource-poor settings. A recent *Lancet* series highlights gaps in mental health services worldwide and introduces a call to action to implement mental health care initiatives in low- and middle-income countries (LMIC) [1-3]. In 2008, the Movement for Global Mental Health, an international coalition aimed at improving treatment for people with mental disorders worldwide, was launched as part of The *Lancet’s* initial call to action [4]. That same year, the World Health Organization (WHO) released a report, titled “Integrating Mental Health into Primary Care: A Global Perspective,” that outlined a strategy to improve and prioritize mental health care. The report focused on the primary areas of training, awareness and sensitization, and service delivery. It recommended integrating mental health into primary care settings to facilitate early, comprehensive, and local treatment for mental disorders [5]. Key strategies to improve mental health care in countries include the following: providing treatment for psychiatric disorders in primary health care settings; increasing patient access to psychotropic medication; educating the public and raising awareness; including families and communities in treatment; establishing national policies and legislation; developing human resources; building relationships with multiple sectors and stakeholders; monitoring community mental health; and supporting research [5,6].

### Framing the problem

The global burden of mental illness has been documented in statistics on overwhelmed health systems, unemployment, limited productivity, and deaths due to mental disorders [6,7]. There are also countless published narratives that detail how stigma and untreated mental illness destroy lives. Every year, approximately 30% of the world’s population develops a mental disorder, and up to two-thirds of those individuals do not receive treatment [6]. Furthermore, the WHO has estimated that mental health problems account for 12% of the Disability-Adjusted Life Years (DALYs) lost [8].

The capacity of health systems to address these problems is restricted in resource-poor settings. Overall, three-fourths of the burden of mental illness (including depression, anxiety, schizophrenia, substance abuse, and dementia) occurs in LMICs [9]. In the context of generally sparse health infrastructure and health systems that focus on communicable and infectious diseases, such as malaria, tuberculosis, and HIV, mental health is often one of the lowest priorities in LMICs. A major obstacle to improving care in LMICs is the lack of practical mental health policies. Such policies are needed to direct comprehensive, coordinated mental health care efforts and facilitate service implementation in local communities [6]. According to recent WHO data, while mental health is mentioned in the general health policy of most African countries (80%), almost half (44%) lack an approved or updated mental health policy [10].

In Senegal, mental illness contributed an estimated seven percent of the country’s disease burden in 2008 [11]. The Senegalese Ministry of Health formulated an official mental health policy that includes promotion, prevention, and advocacy, and the country’s most recent mental health plan was approved in 2006 [12]. Despite these efforts, gaps in implementation persist. Most Senegalese primary care providers have not received formal training on mental health. Official manuals on mental disorders are not available in most clinics, and there is no centralized system to document patient mental health data [10].

Inadequate resources can be one of the primary contributors to funding disparities throughout Africa and other LMIC regions, but lack of finances is not the only cause of poor mental health care. Health care spending in Senegal totals six percent of the gross domestic product [13]. While specific figures are not available, mental health expenditures are presumed to be low. Another challenge to increasing mental health services is limited personnel [14] — e.g., it is estimated that there are only 0.33 psychiatrists and 0.03 psychologists per 100,000 people in Senegal [10]. Other barriers include the fact that specific guidelines for integrating mental health services into primary care settings are often absent, and data on the negative economic impact of mental illness have not been effective in convincing policymakers of the urgency of this issue [15]. Finally, social stigma, which creates negative attitudes about mental illness, and a lack of knowledge often keep those who suffer from seeking treatment [15-17].

### Mental health in Senegal

Senegal is a former French colony in West Africa that gained independence in 1960. It presently has an estimated population of 13 million [18]. The current health system was initially modeled after the French structure. In 1956, the country’s first psychiatric unit was formally established at Fann Hospital in the capital, Dakar.

The results of a post-independence health system assessment revealed that psychiatric services were centralized in Dakar and based on an inpatient asylum model, which aggravated the problems of access to care and inadequate treatment. In 1975, psychiatric villages were established throughout the country in an effort to expand mental health care and facilitate a community model of care [12]. The goal was to establish a psychiatric village in every region in order to deinstitutionalize mental health care, provide psycho-education, and involve families in the treatment process. Currently, only two still exist: one in Ziguinchor (in the southwestern region of the country), which has been transformed into a psychiatric clinic, and one in Tambacounda (in the southeastern region of the country), which is managed by the village community of Botou, about 10 km outside of Tambacounda city. In addition to the psychiatric villages, there are also currently six psychiatric facilities:

• A psychiatric unit at *L’Hôpital Principal* in Dakar, which serves primarily military personnel

• A psychiatric unit at *Hôpital de Saint-Louis*, a regional hospital 300 kilometers from the capital

• *Centre de Sante Mentale” Dalal-Xel,”* a private psychiatric hospital in Thies, 90 kilometers from the capital

• *Thiaroye Psychiatric Hospital,* a private psychiatric hospital in Fatick, 150 kilometers from the capital

• *Centre Psychiatrique Emile Badiane,* a psychiatric center in Ziguinchor, 450 kilometers from the capital

• A small psychiatric unit in the town of Tambacounda, 400 kilometers from Dakar.

Traditional healing practices are common in Senegal and include spiritual and religious ceremonies, divinations, and herbal medicines to treat patients suffering from mental illness and their families [19]. The Ministry of Health does not formally license traditional practitioners; however, there are some mechanisms for collaboration between traditional practitioners and the formal health care structure. For example, there is a traditional medicine bureau at the Ministry of Health, where traditional practitioners are invited to participate in meetings with officials.

Other than WHO disease burden reports, mental health research in Senegal is scant. The limited findings, however, indicate that symptoms of mental illness as well as gaps in health workers’ knowledge and mental health training are significant concerns within the Senegalese health care system. Diop and colleagues found discrepancies between the (higher) rate of psychiatric symptoms based on patients’ self- report and the (lower) rate of patient diagnosis based on health worker screenings at a rural primary health center [20]. This result is consistent with a multi-country study (which included Senegal) that found lack of training among health workers was associated with limited ability to properly recognize psychiatric cases [21].

Findings from two other studies suggest that both Western diagnostic categories and local traditional concepts are sensitive to mental disorders among the population in Senegal. In one study, schizophrenia and hysteria were the top two clinical diagnoses, and interpersonal conflict and daily stresses were the top precipitants of hospitalization in a group of 1,025 adult psychiatric patients [22]. In another study, respondents among the Serer ethnic group were able to discriminate between mentally ill and non-mentally ill individuals in their community using local concepts and idioms of distress, indicating that symptoms of mental illness are salient within local communities in Senegal [23].

### Field examples in Africa

Several field studies have examined the challenges and benefits of integrating mental health services into primary care in Africa. These studies provide a background and helpful insights to understand the context of the current study.

A situational analysis of treatment demand, access, and attitudes toward mental illness at health centers throughout Equatorial Guinea highlights both challenges and strengths of addressing mental health needs. Challenges include a lack of strategic mental health planning and limited programming for the severely mentally ill. Strengths include the country’s well-established primary care system and the government’s commitment to upgrade mental health services [24]. In a similar analysis of mental health integration that compared a middle-income country (South Africa) and two low-income countries (Uganda and Ghana), researchers found major challenges to mental health integration, including inconsistent implementation of mental health policy, too few mental health specialists, and insufficient access to psychiatric medication for severe cases [25].

Addressing health worker attitudes and improving training have also been identified as integral steps to improving mental health care and integrating mental health treatment into primary care. Kapungwe and colleagues [26] found widespread stigma and discriminatory attitudes among primary health workers in Zambia. The authors called for training, awareness programs, and funding to combat stigma and improve understanding of the explanatory models of both patients and health care workers during the training process [26]. In Kenya, a health worker training program that integrated mental health training into existing primary care instruction led to positive outcomes. Specifically, it increased health worker knowledge, improved therapeutic communication with patients and their families, and created new liaisons with the community, including local chiefs [27].

### Rationale for current study

Although policy recommendations on integrating mental health into primary care settings have been published by the WHO, to the best of the authors’ knowledge, they have not been tailored to Senegal’s specific context. In an effort to address the information gap about mental health in primary care settings in Senegal, the current study was undertaken to explore health care workers’ understanding of mental illness, attitudes about integration into primary care, and current activities related to mental health awareness and treatment. The goals were to examine workers’ attitudes and knowledge; identify mental health needs from health care workers’ perspectives; and inform future research, interventions, and policy planning.

## Methods

### General design

This study utilized qualitative key informant interviews.

### Context

Six of eight interviews were conducted in the southeastern region of Kedougou, in the Saraya health district. Two additional health care worker stakeholder interviews were conducted with psychiatrists in Dakar.

Saraya is an isolated, resource-poor area that was established as a new health district by the Senegalese Ministry of Health in 2006. The health staff in Saraya consists of 15 permanent staff members, including a permanent doctor, midwives, nurses, and part-time community health workers (CHW). The district health facilities consist of one health center and six outlying health posts. The population in this district is estimated at over 34,000, with a density of approximately seven persons per square kilometer. Although the majority of the inhabitants are farmers who grow cotton, millet, corn, and peanuts, some also pan for gold in small transient villages (*Diouras*). The main language is Malinké, but Pulaar and Wolof are also spoken. However, French is the primary language used at the health center and surrounding health posts.

This research was conducted as part of a series of community health activities coordinated by the district management team that works on health initiatives such as vaccination campaigns, rapid malaria testing and research, and CHW training.

### Procedures and participants

Ethical approval was obtained through the clinic main coordinator, who is also the district health officer. The study was reviewed and approved by the Health Ministry Committee in Senegal. Verbal informed consent was obtained from all participants. Purposive sampling was used to select respondents with varying levels of experience working in rural primary care settings or familiarity with mental health care in the country. Six key informants from the Saraya health district and two mental health workers/policy stakeholders in Dakar were also recruited. In line with previous research and guidelines on sample size for key informant interviews, we aimed to recruit 8–10 key informants [28,29]. Data saturation, i.e., when no new themes or issues emerged, was achieved after eight participants. Data collection ended at that point. Health workers and stakeholders were recruited in-person by local research assistants and members of the research team. Researchers explained the purpose of the study to the staff at the district health center in Saraya and various stakeholders in Dakar. They then followed up individually with potential respondents to find out if they were interested in participating.

The interviews lasted 45–60 minutes and took place at the health center in Saraya or in participants’ office in Dakar. They were conducted in French and audio recorded, and written notes were taken during the interview. Interview questions focused on knowledge and perceptions of mental illness and the degree to which the participants encountered psychological or psychiatric problems with their patients. Questions were structured so that respondents could also add additional information.

Interviews were comprised of semi-structured exploratory questions that examined health workers’ (physicians, nurses, midwives, community health workers) and policymakers’ awareness and perceptions of mental illness in general and in their specific communities. Questions addressed mental health problems the participants encounter in a clinical setting and their perceptions of how mental illness is identified and treated in the community. The interview questionnaire’s reliability and validity were addressed through consultation among members of the research team in order to establish agreement on the interpretive codes derived from the interviews and determine the suitability of the interview questions. To establish content validity, the interview questions were reviewed in English and French by three of the researchers to assess the breadth of coverage and social and cultural relevance of the questions. Questions were then revised based on the local researchers’ and research assistants’ feedback about social and cultural relevance. Face validity was confirmed by examining the presence of inappropriate responses, which indicate that the questions did not make sense or were not understood. There were very few inappropriate responses.

### Data analysis

Interviews were transcribed, translated into English, and subjected to systematic content analysis to identify response themes. One research team member analyzed the audio and written transcripts using the ATLAS.ti program to organize response categories, which were then reviewed with the other team members. The analysis was driven by an inductive content analysis approach [30], based on grounded theory (i.e., Glaser & Strauss [31]), which allows researchers to inductively develop interpretive codes and further refine the codes to fit the data and reflect respondents’ experiences [30]. The current study’s analysis sought to identify themes, or recurrent concepts, in order to explain broader contextual links [30].

## Results

The participants included two nurses, two midwives, and one community health worker in Saraya; one health post chief nurse from a nearby village; and two psychiatrists working in Dakar. Five participants completed three years of post-secondary training. One attended school up to the eighth grade, and both psychiatrists had medical degrees. Three of the participants (besides the psychiatrists) reported formal instruction on mental illness as part of their training. All of the participants except one were from regions of the country outside of Saraya.

Data coding revealed the following general themes in the interview responses: health workers’ definitions of mental illness (symptoms and causes), community beliefs and practices regarding mental illness, understanding community attitudes toward mental illness, patient problems encountered by health workers, health worker treatment practices, barriers to treatment because of mental illness stigma, mental health training needs of staff, localizing treatment, and government policies and efforts. Key terms that emerged from the analysis include anxiety, depression, schizophrenia, stigma, witchcraft, traditional healers, family and marriage problems, substance abuse, limited availability of psychotropic medications, African culture, resistance to medical treatment, coping with poverty, polygamy, counseling women, midwives’ multiple roles, mental health neglected, limited education, lack of training, health staff isolated, roles, formal and informal counseling strategies, urban and rural differences, and migration.

### Health workers’ definitions of mental illness: symptoms and causes

• Health workers provided a range of explanations about the causes and presentation of mental illness. They described mental illness as any serious psychiatric diagnosis, such as schizophrenia, but also as any condition where someone does not fit the social norms of his or her society, including self-isolation from family. One participant noted, “Mentally ill people live in the street and do things like eat trash”. Another said, “Cutting yourself off from family is a type of mental illness. It is very serious”. Anxiety was described as “a person being agitated and withdrawn,” while participants differentiated psychosis as a more severe illness, defined by inappropriate behavior and inability to care for oneself. For example, one health worker said, “Crazy people hit you and don’t like to wear clothes. Some days they can dress themselves, but usually they cannot”. They reported that mental health problems can be identified by patients’ physical movements, thought processes, and demeanor. One of the participants observed that “someone always appearing happy, walking around and laughing all day long, is not normal”.

• Respondents highlighted poverty, lack of economic development, limited education, drugs, and family problems as causes of sadness and anxiety in the village. One participant pointed out how a lack of appreciation for education among families can lead to mental health issues:

There are many causes, but if you don’t have a high level of education, you don’t have a broad perspective on life. Many families don’t value education or support their children going to school. This may lead you to steal, do drugs - marijuana. That’s why people make [drug] concoctions in the village.

• Another health worker stated, “Ninety-five percent of mental illness is caused by drugs”.

• Sorcery was also identified as a cause of severe mental illness. One participant responded, “Here in Africa, there is also witchcraft or mystique. That is when someone comes across a bad spirit or someone puts a spell on them”. Participants also noted differences between the stressors faced by rural and urban residents that can lead to mental illness. While urban residents struggle to cope with joblessness and under-employment, extreme poverty and social isolation can be sources of distress for villagers. According to one health worker, “some residents don’t have very good rooting in the village - not more than one or two generations, so they feel alone”.

### Community beliefs and practices regarding mental illness

• Health workers reported that individuals in the community have unique cultural and spiritual explanations for mental illness. People in the community often say that “people go crazy because of sickness”, such as a persistent headache, or “after going into the bush and seeing the devil”. Participants reported a common belief among many people (including health workers) that some students “go crazy from school – when they study alone or have to struggle with difficult subjects like math and science”.

• Respondents reported that, in general, community members only recognize severe cases as mental illness. Prior to symptoms becoming severe, people primarily go to *marabouts* (Muslim clerics who use Islamic religious traditions in their healing practices) and traditional healers (exorcists, herbalists) for treatment of a range of psychosocial and psychiatric symptoms. They typically only see a medical doctor after the symptoms become worse. By the time many severely ill patients are brought to the clinic, “they have been ill for at least 1 year and have been treated by a traditional healer”, noted one respondent. Another participant reported, “Some patients are brought by their families when they are very agitated and are then given benzodiazepine to calm down”. Another stated, “Mentally unstable people don’t come here – they only come here if they’re sick. The psychotic patients never come here”.

### Understanding community attitude to mental illness

• According to the psychiatrists interviewed, two important factors must be considered in understanding the community’s attitude about psychiatric treatment for mental illness. One is the feeling among many people that the discipline of psychiatry “was forced upon Africa by Europeans and that many people want to re-affirm their cultural roots by addressing psychological and social problems using traditional means”. Secondly, the community’s unique cultural interpretation of symptoms leans toward “externalized explanations of symptoms and illness, such as witchcraft and evil spirits”.

### Patient problems encountered by health workers

• Health workers reported that they often encounter repatriated immigrants who return from abroad with social, psychological, and financial problems. Respondents also acknowledged that psychological issues underlie a number of patient physical symptoms they treat, especially vague complaints and gastrointestinal problems. Other patient problems that health workers reported include unwanted pregnancies and social and emotional problems experienced by women who have reached menopause. Noting that there is a mental health aspect associated with most of the patient complaints, one health worker said, “Many people have psychological and mental health problems that need to be dealt with before they turn to something worse”.

• Midwives reported dealing with psychological and emotional distress “due to women’s situations” among female patients. They are often privy to female patients’ fears and problems, including relationship conflict, polygamous marriages, and sexually transmitted infections (STIs). According to one midwife, “the majority of families in the village practice polygamy. It is common for two wives [who are in a polygamous marriage] to have problems with each other or the husband”. Another midwife added, “Sometimes when a woman comes in with an STI, health workers have difficulty following the standard medical practice of treating all of the partners [i.e., husband and all the wives] because the woman is afraid to admit to her husband that she has an STI”. The midwives reported engaging in a lot of informal counseling with women facing these situations.

### Stigma and barriers to treatment

• Respondents noted that stigma about mental illness—from medical doctors as well as the general public—is widespread in the community. Additional barriers that were noted include limited availability of neuropsychiatric medications, an insufficient number of mental health specialists, and social changes in the society. One participant observed that, “there are more mentally ill people wandering the streets in the urban cities because of changes in the family structure. We need to prevent these problems in the village”. Citing the fact that not all regions of the country have hospitals with psychiatric units, another health worker said, “Mental illness is neglected here in the village”.

### Mental health training needs of staff

• The health workers stated that, despite their familiarity with the community and the types of problems patients experience, they desire more training on recognizing and addressing mental illness, especially severe psychiatric diagnoses. They also expressed the need for general professional support. According to one respondent, “sometimes we can feel a bit isolated and overwhelmed here at the health center. We are not from here and we have to see many patients”.

### Localizing treatment

• The *N’dep* ritual was described as an example of an approach to localizing mental health treatment and making it more culturally relevant. The psychiatrists interviewed described N’dep as a traditional Wolof ritual that has been adapted as a communal treatment in some parts of Senegal. In some psychiatric hospitals, N’dep has been used as a form of therapy where the group is headed by a patient and held in a traditional group circle called a *penc,* named after a tree that symbolizes community support. The custom calls for community members to sit together to discuss important issues. One participant stated that “an approach that includes the community helping care for patients is best for the Senegalese people”. Respondents noted that a focus on local knowledge is important because even for Senegalese doctors within the country, it is not always possible to understand the dynamics of each region and locality.

### Government policies and efforts

• Respondents reported that current efforts to improve mental health care include creating an instructional manual for health workers, planning a school to train nurses to treat mental illness, and collecting statistics on the prevalence of various disorders. However, funding is a problem, and the fact that patients do not tend to use existing health structures makes collecting diagnostic data difficult. There have also been plans to coordinate mobile visits to patients’ homes in rural areas, but funding and planning have been significant obstacles. According to one respondent, “there is a national mental health plan, but the activities are not planned in a coordinated way”.

## Discussion

These data provide insight into the intersection of policy, health worker perspectives, and community attitudes on mental health treatment in rural Senegal. The interview responses reflect some of the recent discourse in global mental health survey research and case studies of mental health integration efforts in Africa. An important finding is that participants presented a psycho-social-cultural understanding of mental illness in rural Senegal that can be used to further refine efforts to integrate mental illness treatment into primary care settings.

The respondents in this study brought up challenges that have been discussed in previous research, such as inconsistent and uncoordinated policies, and insufficient training and resources to treat severe cases [25]. Similar to conclusions drawn from research in Equatorial Guinea, the findings here point to the potential strength of a well-established primary care system [24], albeit one that is overly centralized in Senegal. For example, the model in Senegal of having district health facilities that are associated with smaller health posts could provide a strong network for localizing formal health worker training and community awareness activities.

These findings are also consistent with previous work on the impact of stigma (from both community members and health workers) as a barrier to service utilization and integration of mental health treatment [26]. A strategy recommended by researchers to overcome this stigma is to implement targeted awareness and sensitization campaigns [26,32]. Another important barrier to treatment is limited funding. Given health worker reports of limited resources, it is important to consider that additional reasons that patients in Saraya and surrounding villages do not seek help include the cost associated with care and patients’ awareness that the facility may not be fully equipped to treat them (which is a function of national funding problems).

Understanding and integrating the cultural context into care is critical for buy-in and commitment, and holds promise as a pathway to better integration of mental health care. Respondents’ observation that the population may reject psychiatric treatment as “un-African” is significant for developing strategies that are congruent with local contexts and beliefs [32,33]. This may call for more focused efforts to recognize parallel health systems and consult with traditional healers about common patient problems and access to patients. Admittedly, such collaboration would be a difficult task. While communities may prefer traditional healers and treatments, poverty and rapid social changes make it problematic for traditional systems to carry the weight of tending to the mentally ill. Contemporary health infrastructure and training are clearly necessary to address patient needs. A comprehensive approach, that takes into account the role of traditional healers [24] while also prioritizing spending and training, should be part of future planning. For example, the use of the N’dep group, cited by respondents as a way to integrate culture into existing treatment approaches, provides a useful model of localization.

One key conclusion that can be drawn is that health care workers in rural Senegal can be trained to do formal and informal mental health screening due, in part, to their strong knowledge of the local cultural context. Previous research demonstrates that even minimal training for counselors in their own communities can be effective in addressing and treating anxiety and depression in low-resource settings [34]. In Kenya, even limited training for primary health care workers resulted in measurable improvements in health worker knowledge of mental illness and therapeutic relationships with patients and the community [27]. In the context of the current study, training would also be important in providing health workers with accurate information on the symptoms and causes of psychiatric illness.

As the respondents highlighted, there is clear evidence that mental health care is neglected, particularly in remote regions of the country. Integrating mental health care into primary care settings throughout the country is an important strategy to address the disparities and neglect. Some of the primary goals of integration include capacity-building, training non-specialists, raising awareness, and sensitizing the community. A number of investigators [32,33] have recommended that countries align policies with their social and cultural realities [32] to reduce stigma and raise awareness. These goals are often recommended where mental health policy is non-existent or scant [24], such as in Senegal. The current findings support such policy recommendations and goals.

However, previous studies have identified a number of challenges to achieving these aims, including lack of specialists, limited access to medication, and poor implementation policies [25]. The participants in this study recognized similar challenges in their setting and acknowledged their need for more knowledge and training about mental illness. Furthermore, making mental health one of the national health priorities is also necessary to improve mental health care in the country. Research on mental health care in LMICs confirms that policy needs to be clearly articulated and consistently applied, and that spending should be prioritized and focused [5,6,13].

The participants in this study pointed out the relationship of poor mental health to larger social factors, including poverty, migration, and education. The role of these global development factors calls for the involvement of multiple sectors in improving mental health care. Therefore, NGO’s, government and international aid agencies, and the private sector can play a role in crafting mental health policy and strategies.

### Implications

The science and practice of integrating mental health into the primary care setting in this resource-poor country need to be given priority in order to lower the disease burden of mental illness. Capacity building may include engaging various stakeholders and partners—Ministry of Health, the medical faculty at the country’s largest university (Cheikh Anta Diop University)—and expert researchers and practitioners in the country. Training community health workers and mid-level medical personnel, such as nurses and midwives, particularly in rural areas, is a feasible way to better meet the mental health needs of the community.

A practical approach toward developing a comprehensive primary care treatment plan could include basic patient mental health screening by the health staff and CHWs. Although patients may go directly to health centers or hospitals when the problem is severe enough, the CHWs are often the first point of contact with the health system. Plans for prevention and treatment need to address stigma, cultural beliefs, patients’ use of parallel health systems, and availability of medication. Additionally, plans should be developed to conduct small-scale awareness/sensitization programs that are specifically tailored to rural communities.

Given that patients primarily go to marabouts and traditional healers for treatment either before or while seeing a medical doctor, it would be worthwhile for policymakers and researchers to explore how to further integrate traditional healing practitioners into formal mental health care. Finding ways to capitalize on the population’s strong cultural values and their confidence in traditional healers could be a possible avenue to expand mental health care where there are currently gaps, particularly in rural regions. Initial efforts should focus on assessing and documenting the current scope of traditional practitioners’ treatment. Further efforts should explore the feasibility and challenges of formal collaborations between traditional and medical practitioners, with an emphasis on traditional healers’ roles in assessing and alleviating psychosocial distress among patients.

The need for training and support should not be overlooked. Health workers seem to be carrying out multiple roles that might be overwhelming in isolated settings. Their experience of dealing with mental health issues while also being aware that they are not fully equipped to address those issues can lead them to experience work burnout. Health worker wellbeing is critical to the successful implementation of mental health programs and should also be prioritized within the health care system.

Additionally, these findings point to several possible areas for future research, including the following:

• Community definitions of mental illness, including cultural perceptions and conceptualizations

• Community uses of traditional healers in treatment of mental disorders

• Prevalence of mental disorders, such as depression, anxiety, psychosis, and trauma

• Specific gender issues—differences in assessment, treatment, and outcomes for women vs. men

• Awareness related to women’s issues, such as menopause and other reproductive health concerns

• Child and adolescents mental health

• Systemic challenges and opportunities for integrating mental health and primary care and increasing effectiveness of psychosocial approaches.

This study’s findings help to establish a platform for more extensive qualitative and quantitative research that addresses the gaps in treatment. The small sample size suggests that additional studies are needed to examine other important topics and generalize these initial findings.

## Conclusions

This small exploratory study is significant in that it describes several aspects of the contemporary mental health landscape in a region where there has been limited mental health research. An important finding is that health workers presented a psycho-social-cultural understanding of mental illness in rural Senegal that can be used to further refine efforts to integrate mental illness into primary care settings. Health workers’ strong knowledge of the local cultural context is a strength that can be used for further program and policy planning. Recommendations for developing a comprehensive mental health primary care treatment approach include patient mental health screening, incorporating rural villagers' attitudes and beliefs about mental illness, and utilizing community health workers—who are often a first health contact for many in the community—to work with the medical staff to identify mental health problems.

## Competing interests

The authors declare that they have no competing interests.

## Authors’ contributions

NM conceived the study, analyzed and interpreted the data, and wrote the manuscript. NM, DB, and YN developed the research design and interview protocol. NM and DB conducted the interviews. DB, YN, and IB made substantive contributions to data interpretation and were integrally involved in revising the manuscript. All authors read and approved the final manuscript.

## Authors’ information

NM, PhD; YN, MD; DB, MD, MPH; IB, MD.
